# Casein phosphopeptide amorphous calcium phosphate and universal adhesive resin as a complementary approach for management of white spot lesions: an in-vitro study

**DOI:** 10.1186/s40510-022-00404-9

**Published:** 2022-03-21

**Authors:** Neven S. Aref, Maha Kh. Alrasheed

**Affiliations:** 1 Department of Dental Biomaterials, Faculty of Dentistry, Mansoura University, El Gomhoria St, Mansoura, 35516 Egypt; 2 Department of Basic Oral and Medical Sciences, College of Dentistry, Qassim University, Buraydah, Saudi Arabia; 3 College of Dentistry, Qassim University, Buraydah, Saudi Arabia

**Keywords:** CPP-ACP, Enamel demineralization, Resin infiltration, Universal adhesive resin, White spot lesion

## Abstract

**Background:**

White spot lesion (WSL) is the most common consequence during and after orthodontic treatment. This study was conducted to investigate the ability of casein phosphopeptide amorphous calcium phosphate (CPP-ACP) coupled with universal adhesive resin to treat white spot lesions.

**Material and methods:**

Forty-five extracted premolars were sectioned to create 90 specimens. Seventy-five specimens were demineralized to generate artificially created WSLs. Different strategies have been applied for the management of the artificially created WSLs. Six experimental groups were employed: Group I: sound enamel (control), Group II: demineralized enamel (artificially-created WSLs), Group III: ICON resin-treated WSLs, Group IV: CPP-ACP-treated WSLs, Group V: universal adhesive resin-treated WSLs, and Group VI: CPP-ACP followed by universal adhesive resin-treated WSLs. Assessment of color stability using a spectrophotometer, surface microhardness using a Vickers tester, and surface roughness using a profilometer was done. The surface topography of representative specimens from each experimental group was inspected using a scanning electron microscope. Collected data were analyzed using one-way ANOVA followed by Tukey’s post hoc test at *p* ≤ 0.05.

**Results:**

White spot lesions treated with CPP-ACP and subsequently coated with universal adhesive resin (Group VI) exhibited a significantly lower Δ*E* than both CPP-ACP (Group IV) and universal adhesive resin-treated (Group V) groups (*p* ≤ 0.05), but it was not significantly different from the ICON resin-treated group (Group III). For surface microhardness, WSLs treated with CPP-ACP and consequently coated with universal adhesive resin (Group VI) recorded the highest mean that was significantly different from both ICON resin (Group III) and universal adhesive resin-treated (Group V) groups (*p* ≤ 0.05). All the tested strategies (ICON resin, CPP-ACP, universal adhesive resin, and CPP-ACP followed by universal adhesive resin) significantly lowered the surface roughness of the WSLs (*p* ≤ 0.05), while no significant difference was detected among them.

**Conclusions:**

Combining a considerable caries remineralizing program using CPP-ACP with subsequent universal adhesive resin infiltration could be a promising approach to manage WSLs efficiently through increasing surface microhardness and restoring esthetic while developing a smoother surface.

## Background

Fixed orthodontic appliances are usually accompanied by enamel demineralization or white spot lesions (WSLs) [1]. The appearance of these lesions is mainly caused by the prolonged accumulation of dental plaque and bacterial colonization around the orthodontic brackets, under the arch wire, or between the brackets and the gingival margin with difficult tooth cleaning [2]. The self-cleaning mechanism of the oral musculature and saliva is prohibited by the irregular surface of the brackets, wires, and bands. Consequent active white spot lesions may develop enamel cavitation if left untreated [3].

Microabrasion therapeutic option is best appropriate for very superficial lesions, less than 0.2 mm. Deeper lesions always cause concave tooth surfaces [4]. Direct and indirect restorations produce good and predictable results as well, but they should be limited to cavitated lesions [5].

Great attention has been paid to non invasive therapy of enamel carious lesions with remineralizing agents such as fluoride and casein phosphopeptide-amorphous calcium phosphate (CPP-ACP), or the use of therapeutic sealants for occlusal lesions [5]. Fluoride treatment results in a less soluble, acid-resistant fluorapatite on the enamel surface. Fluoride-containing varnishes, toothpastes, mouth rinses, and gels are examples of fluoride delivery. Although fluoride's capacity to remineralize enamel is well established, there are little data to support its use in post-debonding white lesions [6, 7]. Application of CPP-ACP, or bioactive glass, are more recent techniques to minimize the white spot formation [8, 9]. They may be utilized before or after the fixed appliances are bonded. These preventive strategies have been employed to avoid the initiation, to arrest or reverse the progression, or to mask the WSLs. They are confined to remineralization of superficial white spot lesions and cannot entirely prevent their development [10, 11]. Even though these products can aid in remineralization, the esthetic mostly remains impaired [12–14]. Additionally, they are not always successful because they involve patient compliance as well as a change in detrimental habits, and many patients stop therapy before it is completed [4].

Nanomaterials with anti-adhesive properties have been used to manage oral biofilm and minimize demineralization around brackets [15]. Silver nanoparticles, nano-hydroxyapatite, and titanium dioxide nanoparticles were the most commonly investigated for use in nano-filled orthodontic adhesives [16–18]. Although the application of nanoparticles in orthodontics has had promising results, there is a lack of studies determining whether nano-filled adhesives have a long-term effect on the prevention of enamel demineralization during orthodontic therapy [15].

As a modern and more conservative approach, infiltration of the WSL with a hydrophobic low-viscosity composite resin has been developed, reflecting the minimally invasive approach. This resin-based treatment infiltrates the body of the lesion via capillary forces, plugging the pores between the pathological crystals of the enamel and forming a diffusion barrier on the surface of the enamel and within its deeper layers, obstructing acid entry pathways and so hindering the expansion of lesions. This allows reflection of light in a comparable manner to the neighboring sound enamel [19, 20].

The treatment approach is highly dependent on the depth and extent of the single lesion. If they are maintained to a minimum extent, infiltration of the lesion with a low-viscosity resin is the best option. The resin infiltration procedure is proper, minimally invasive, painless, speedy and instantly improves the patient's oral esthetic. Despite being the most conservative method, resin infiltration is far from ideal because the lesion is masked rather than eliminated, and subsequent follow-ups are necessary to assess and maintain esthetic stability [21].

Universal adhesives, also known as multi-mode adhesives, are the newest type of adhesive. These adhesives contain an acidic functional monomer called 10-methacryloyloxydecyl dihydrogen phosphate (MDP), which generates surface micro-retention and chemical interactions with calcium in the tooth hydroxyapatite [22].

Combining the minimally invasive strategy (esthetic-improved strategy) through resin infiltration with a considerable preceding caries remineralization program (reversing lesion-based strategy) may give therapeutic benefits plus extremely reduce the long-term restorative demands and expenses, thus supporting the concept of a minimally invasive treatment option [23, 24].

CPP-ACP and universal adhesive resin may act synergistically to extensively remineralize white spot lesions and restore esthetic. To date, there is no evidence that treatment of WSLs with CPP-ACP remineralizing agent with subsequent universal adhesive resin infiltration may offer an alternative and effective treatment modality for WSLs. The research hypothesis is based on finding a definitive answer to the query: could casein phosphopeptide amorphous calcium phosphate combined with subsequent universal adhesive resin infiltration be a complementary approach for the management of white spot lesions? The null hypothesis was that casein phosphopeptide amorphous calcium phosphate coupled with consequent universal adhesive resin infiltration would not be able to provide an optimal treatment for white spot lesions.

## Materials and methods

### Materials used in the study

1. Hydroxy ethyl cellulose (HEC) powder (Sigma 09368, GmbH, Rieddstr., Steinheim, Germany)
2. DL-Lactic acid (Sigma 69785, GmbH, Rieddstr., Steinheim, Germany).
3. ICON resin infiltrant (DMG Dental Materials, Hamburg, Germany).
4. Casein phosphopeptide amorphous calcium phosphate (CPP-ACP) remineralizing paste (GC Corporation, Tokyo, Japan).
5. Universal adhesive resin (GLUMA Bond Universal, Heraeus Kulzer).

### Teeth selection and experimental design

**Fig. 1:**
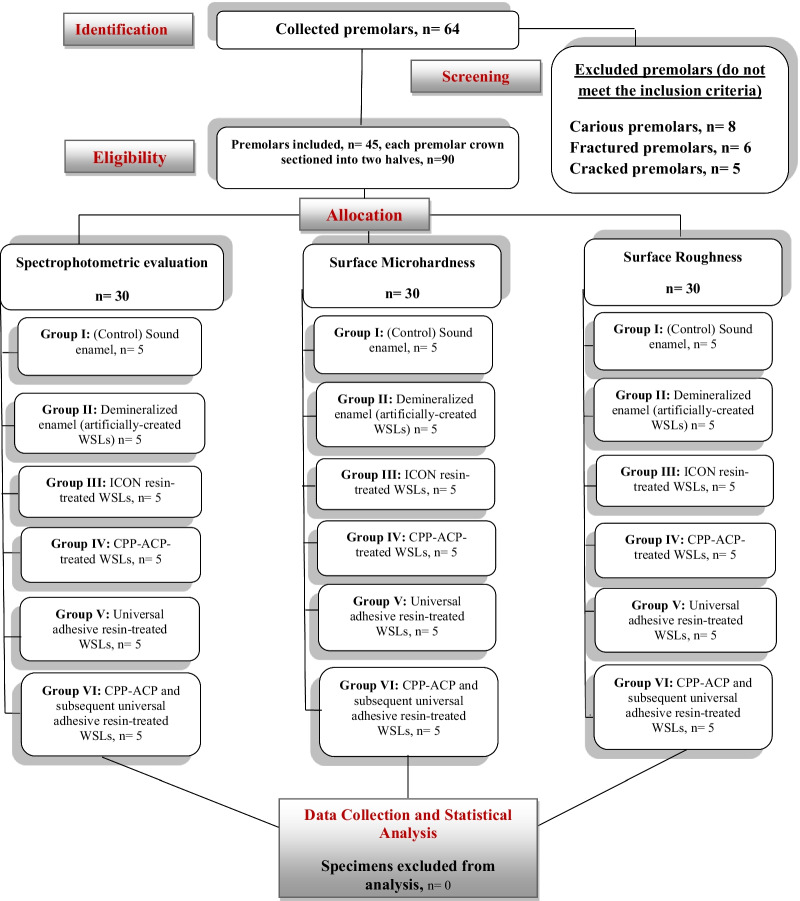
Consort flow diagram

The study was designed according to the Consolidated Standards of Reporting Trials (CONSORT) statement (Fig. 1). Forty-five sound premolars extracted for orthodontic reasons were collected from the outpatient clinic, College of Dentistry, Qassim University (In accordance with the ethical standards of the institutional research committee at Qassim University, ethical approval no. EA/F-2020–3012).

The teeth were washed with distilled water to remove any blood or soft tissue remnants and then stored in an aqueous 0.1% Thymol solution. The teeth were cleaned using a spoon excavator and a tooth brush to remove any soft tissue remnants. The teeth were dried and inspected under a stereomicroscope (Olympus, Japan, SZ-PT model) at 10× magnification to ensure the absence of stains, demineralization, hypoplasia, fluorosis, or enamel cracks.

The roots of the selected teeth were cut away at the cementoenamel junction, and the crown of each tooth was sectioned buccolingually into two halves using a cutting machine (Isomet 4000 microsaw, Buehler, USA), producing 90 specimens. The specimens were randomly divided into six groups as follows: Group I: sound enamel (control), Group II: demineralized enamel (artificially-created WSLs), Group III: ICON resin-treated WSLs, Group IV: CPP-ACP-treated WSLs, Group V: universal adhesive resin-treated WSLs, and Group VI: CPP-ACP followed by universal adhesive resin-treated WSLs.

The enamel surface of each specimen was partially covered with acid-resistant nail polish, leaving an investigational window of about 4 × 3 mm. At 37 °C, white spot lesions were artificially-created on specimens of groups II, III, IV, V, and VI using an acidic hydroxy ethyl cellulose (HEC) gel of pH 4.95–5 and were kept for 10 days with renewal of the demineralizing gel every 3 days.

Acidified HEC gel was prepared by dissolving HEC powder at a ratio of 140 g/L into distilled water, forming a viscous solution. The pH was adjusted using a solution containing 0.05 M lactic acid. The solution was continuously stirred for 30 min until the HEC was partially hydrolyzed. The resultant HEC gel was poured into a container and placed into an incubator at 37 °C for about 24 h. Specimens from each experimental group were placed into a sealable container, and the fully hydrolyzed HEC gel was poured over them. Specimens were removed from the acidified gel after the decalcification process was completed. Washing of the specimens with distilled water and drying with compressed air for 10 s.

### Treatment of the artificially-created WSLs

For group III, ICON resin was applied and cured after hydrochloric acid etching of the specimens and ICON dry application according to the manufacturer's instructions. For groups IV and VI, a thin coat of CPP-ACP paste was applied with a micro-brush to the flat demineralized enamel window for 3 min every day and left untouched. The specimens were then cleaned for 20 s in distilled water and stored in distilled water until the next application. This process was repeated for 6 weeks. For group VI, after the CPP-ACP remineralizing program had been completed, specimens were treated with the universal adhesive resin. The adhesive resin (Group V and VI) was applied and cured according to the manufacturer's instructions. Specimens were stored in distilled water at 37 °C for 48 h before testing.

### Spectrophotometric evaluation

A spectrophotometer (Vita Zahnfabrik H. Rauter GmbH & Co. KG, Bad Sackingen, Germany) was used for the measurement of the color values (L*a*b*) according to the manufacturer's instructions. Readings obtained represent the measured CIE L*, a*, and b* for each specimen, which were compared to that of the control specimen (sound enamel specimen), and the color difference (Δ*E*) was calculated by the following formula [25]:

$$\Delta E = \left[ {\left( {\Delta L*} \right)^{2} + \left( {\Delta a*} \right)^{2} + \left( {\Delta b*} \right)^{2} } \right]^{1/2}$$

where L* represents the color value, a* and b* represent chromaticity. Δ*E* value equal or larger than 3.3 was considered a clinically recognizable color change.

### Surface microhardness evaluation

The indenter of a Vickers microhardness tester (Micromet II, Buehler, Lake Bluff, IL, USA) was used to make three indentations on each experimental enamel surface window, each 100 μm apart, with a static force of 200 g for a 15-s dwell time. The mean Vickers hardness number (VHN) was calculated as the average of the three values and expressed in kg/mm^2^.

### Surface roughness assessment

A profilometer (Mitutoyo, Sakado, Japan) was used to measure the surface roughness of the specimens to an accuracy of 0.01 mm. The surface roughness cutoff value was 0.25–2 mm, and the stylus’ traversing range was 3 mm. The diamond tip radius was 2 μm, the tip angle was 60°, with the measuring force of 0.75 mN and a velocity of 0.5 ms^−1^. Three measurements were recorded for each specimen, and average roughness values (Ra) were calculated and stated in μm.

### Surface topography

The surface of representative specimens from the different experimental groups was inspected by a scanning electron microscope (JEOL, JSM-6510LV, Japan) at a magnification of ×2000.

Data analysis was conducted utilizing the "Statistical Package for Social Sciences (SPSS) version 20.0" (SPSS Inc., Chicago, IL) using one-way ANOVA and subsequent Tukey’s post hoc tests for pairwise comparison with a significance factor of *α* = 0.05.

## Results

**Fig. 2:**
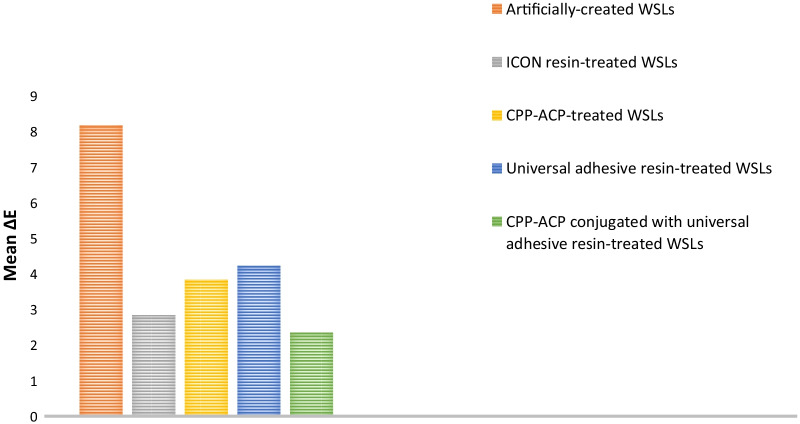
Color difference results of the investigated groups

Table 1 denotes means, standard deviations, and Tukey’s analysis of the color difference among the investigated groups. Demineralized enamel exhibited the highest mean value (8.18 ± 0.43), while the demineralized enamel treated with CPP-ACP followed by a subsequent coat of universal adhesive resin had the lowest mean value (2.35 ± 0.21). ANOVA stated a significant difference among the different groups (*p* ≤ 0.05). At *p* ≤ 0.05, white spot lesions treated with CPP-ACP coupled with a subsequent universal adhesive resin and those treated with ICON resin were significantly different from WSLs treated with CPP-ACP and those treated with universal adhesive resin. On the other hand, no statistical difference was detected between CPP-ACP coupled with a subsequent universal adhesive resin-treated lesions and ICON resin-treated ones. A graphical presentation of the color difference results is shown in Fig. 2.

**Table 1:** Means, standard deviations, and Tukey’s analysis of the color difference of the studied groups (*p* < 0.0001)

Groups	Color difference (Δ*E*)
	Mean ± SD
II (Artificially created WSLs)	8.18^a^ ± 0.43
III (ICON resin-treated WSLs)	2.83^c^ ± 0.23
IV (CPP-ACP-treated WSLs)	3.84^b^ ± 0.75
V (Universal adhesive resin-treated WSLs)	4.23^b^ ± 0.26
VI (CPP-ACP and universal adhesive resin-treated WSLs)	2.35^c^ ± 0.21

Means with the same superscript letter are not significantly different at *p* ≤ 0.05

**Fig. 3:**
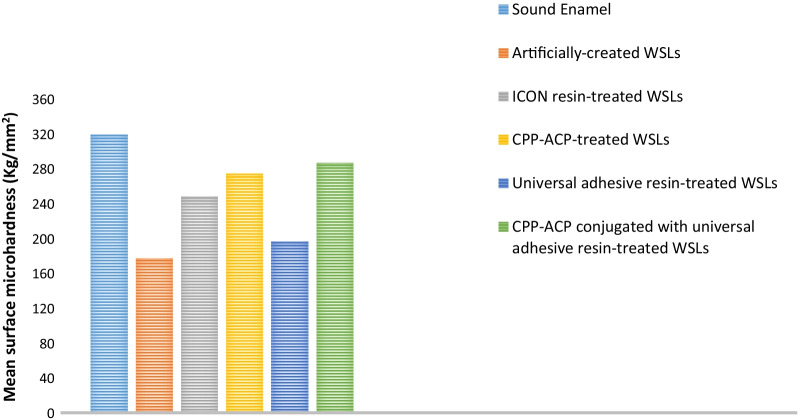
Surface microhardness results of the investigated groups

Table 2 represents means, standard deviations, and Turkey’s analysis of the surface microhardness (kg/mm^2^) and surface roughness (μm) of the considered groups. For surface microhardness, sound enamel showed the highest mean value (319.2 ± 3.16), while demineralized enamel had the lowest value (177.8 ± 2.2). ANOVA revealed a significant difference among the studied groups (*p* ≤ 0.05), in which all groups were significantly different from each other. However, no significant difference was detected between WSLs treated with CPP-ACP and those treated with CPP-ACP coupled with universal adhesive resin at *p* ≤ 0.05. The surface microhardness results are graphically presented in Fig. 3.

**Table 2:** Means, standard deviations, and Tukey’s analysis of the surface microhardness and surface roughness of the studied groups (*p* < 0.0001)

Group	Surface microhardness (kg/mm^2^)	Surface roughness (μm)
I (Sound enamel)	319.2^a^ ± 3.16	1^c^ ± 0.02
II (Artificially created WSLs)	177.8^e^ ± 2.2	2.35^a^ ± 0.21
III (ICON resin-treated WSLs)	248.3^c^ ± 3.03	1.65^b^ ± 0.16
IV (CPP-ACP-treated WSLs)	274.48^b^ ± 3.54	1.83^b^ ± 0.08
V (Universal adhesive resin-treated WSLs)	196.78^d^ ± 5.35	1.73^b^ ± 0.07
VI (CPP-ACP and universal adhesive resin-treated WSLs)	286.82^b^ ± 4.57	1.56^b^ ± 0.1

Means with the same superscript letter in each column are not significantly different at *p* ≤ 0.05

**Fig. 4:**
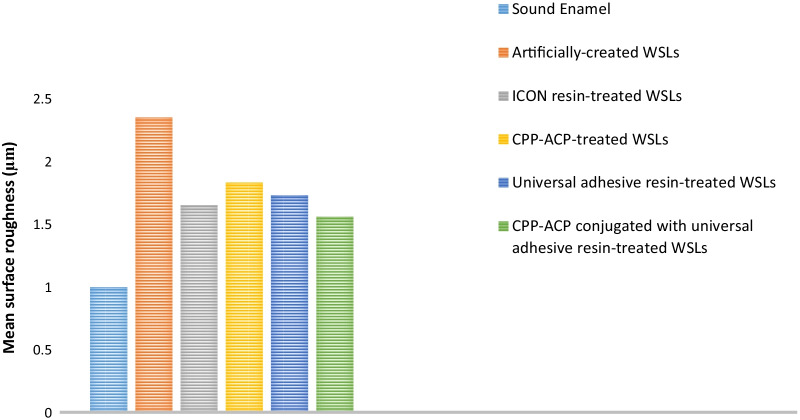
Surface roughness results of the investigated groups

In terms of surface roughness, demineralized enamel displayed the highest mean value (2.35 ± 0.21), while sound enamel presented the lowest value (1 ± 0.02). ANOVA identified a significant difference among the studied groups (*p* ≤ 0.05). No significant difference was observed among WSLs treated with ICON resin, CPP-ACP, universal adhesive resin, and the one treated with CPP-ACP followed by a further covering of universal adhesive resin at *p* ≤ 0.05. Conversely, all formerly mentioned treatment approaches were significantly different from artificially-created WSLs. The surface roughness results are graphically expressed in Fig. 4.

**Fig. 5:**
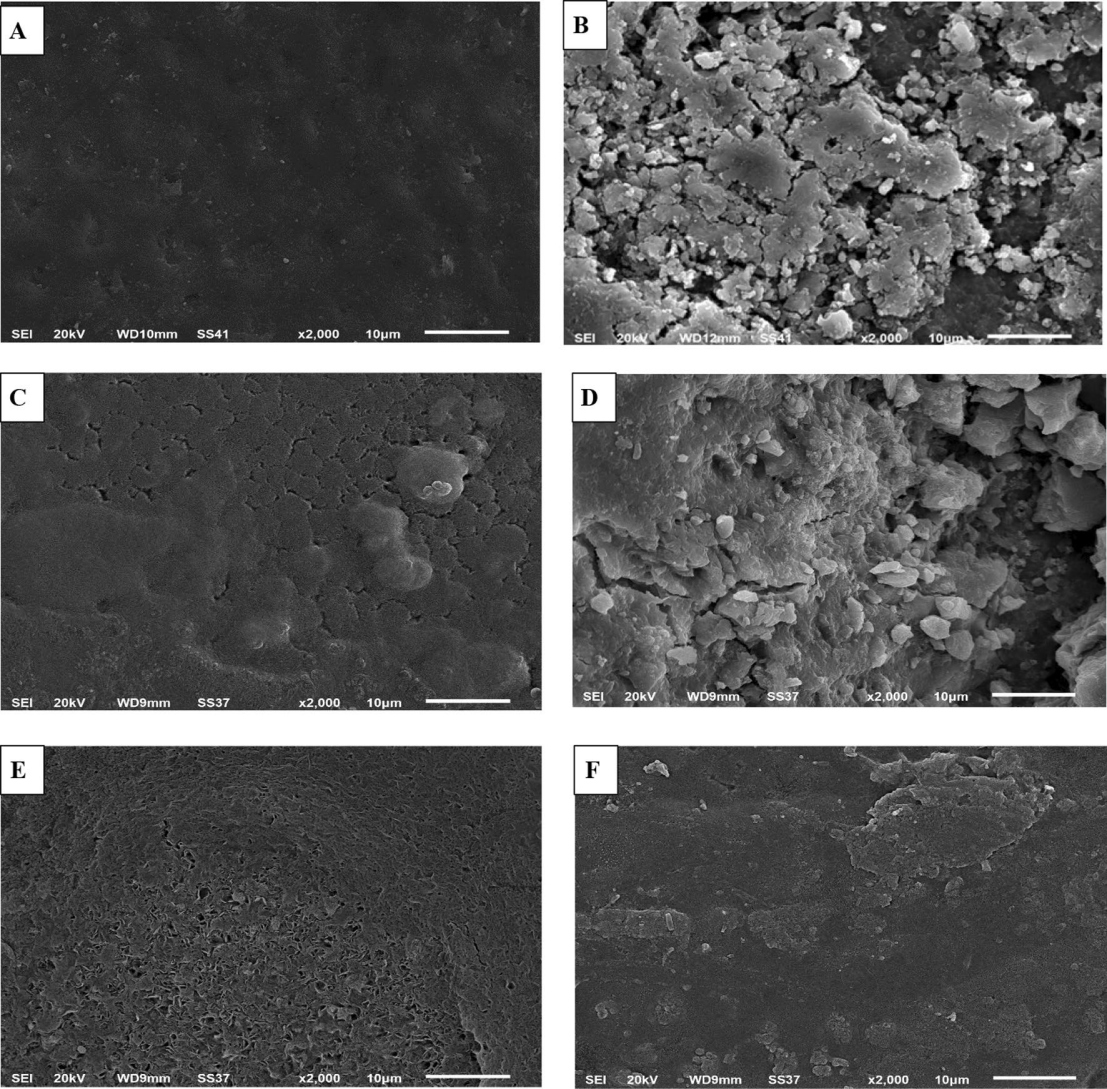
Scanning electron micrographs (× 2000 magnification) of the different experimental groups; **a** Sound enamel, **b** Artificially-created white spot lesion (demineralized enamel), **c** ICON resin-treated white spot lesion, **d** CPP-ACP-treated white spot lesion, **e** Universal adhesive resin-treated white spot lesion, **f** CPP-ACP with subsequent universal adhesive resin-treated white spot lesion

The scanning electron micrographs are shown in Fig. 5. Sound enamel (Fig. 5a) showed a smooth, homogenous surface with fish scales' characteristic look. The demineralized enamel surface (Fig. 5b) displayed observable pitting, discontinuity, and irregularity of the surface, denoting extreme roughness due to destruction of the enamel rods and dissolution of enamel crystals during demineralization. On application of ICON resin (Fig. 5c), obliteration of the enamel rods was observable, revealing a more even and homogenous surface compared to the remarkable roughness of the preceding demineralized enamel. The CPP-ACP-treated enamel surface indicated the ability of CCP-ACP paste to restore a uniform, thick, homogenous, and compacted surface layer with a globular structure and decreased depth of the inter-prismatic cavities formed by demineralization (Fig. 5d). Figure 5e shows the ability of the universal adhesive resin to mostly infiltrate the demineralized enamel surface, forming a homogenous coat of intermingled network. The demineralized enamel treated with CPP-ACP conjugated with subsequent universal adhesive resin showed the most consistent and least porous surface among WSLs treated with other different protocols, representing the ability of the resins to occlude the remaining inter-prismatic cavities left after CPP-ACP treatment, as shown in Fig. 5f.

## Discussion

Resin infiltration has been widely used to treat white spot lesions because of its ability to restore esthetic promptly. However, because of its potential to mask the lesion rather than remineralize it, its use as an optimal treatment option is controversial and is a cause for concern [21]. So, this study investigated the ability of CPP-ACP coupled with universal adhesive resin to remineralize the white spot lesion and restore its esthetic, proposing an optimal treatment modality.

The findings of the study endorsed the rejection of the null hypothesis. The WSL treatment protocol based on using CPP-ACP paste to remineralize the lesion followed by a universal adhesive resin coat to mask the lesion was a comparable or even better treatment approach than the most commonly used approaches like ICON resin infiltrant or CPP-ACP remineralizing agent separately.

Management of white spot lesions via CPP-ACP alone was not able to restore the esthetic to a non-observable degree as (Δ*E* ≥ 3.3). On pairing this remineralizing program with universal adhesive resin, the results were very promising (Δ*E* = 2.35). The universal adhesive alone exhibited the least ability to enhance esthetic (Δ*E* = 4.23) among the investigated strategies.

The bioactive substance generated from casein milk protein, casein phosphopeptide amorphous calcium phosphate, can function as a reservoir of bio-available calcium and phosphate, allowing their precipitation on the demineralized enamel surface and so considerably improving remineralization [26, 27]. It has been reported that CPP-ACP is able to fill the subsurface enamel pores, decreasing the refractive index difference between the porous demineralized enamel and the sound enamel, thus improving the translucency of the white spot lesion [28]. This is consistent with the finding by Anggani et al., which concluded that CPP-ACP application enhanced the esthetic of white spot lesions because of CPP-ACP's ability to fill the subsurface enamel pores. This supporting study applied CPP-ACP once/a day for 14 days [29]. Alternatively, our finding is in disagreement with a different study that stated the inability of CPP-ACP application to enhance the esthetic of post-debonding white spot lesions and correlated that to the short duration of CPP-ACP application, which was only once with follow-up after 6 weeks [30]. So CPP-ACP effectiveness seems to be treatment duration and follow-up time-dependent. One more investigation recommended the daily application of CPP-ACP and considered it more effective in regression and esthetic improvement of post-debonding WSLs than fluoride-containing pastes and rinses [31]. Also, the results of a 12-week clinical study show that twice daily topical applications of 10% CPP-ACP paste as an important contributor to a standard oral hygiene program, such as fluoridated dentifrice, antimicrobial mouthwash, and xylitol chewing gum, improve the esthetic and remineralization of white spot lesions significantly [32].

Variations in study design, duration of the treatment protocol application, variances in the activity and severity of lesions, and probable pathological distinctions between orthodontic and non-orthodontic WSLs may all contribute to differences in CPP-ACP efficiency and in-vivo recommendations [33, 34]. This may explain the variability among different studies’ assumptions.

On treating the WSLs with either ICON resin or universal adhesive resin, the esthetic was improved, but not to the same grade with a statistically significant difference in between. Generally, the resin has the same refractive index as enamel and, on application, it infiltrates the pores, obliterating them and thus minimizing the difference in refractive index between the pores and the neighboring sound enamel surface to the point of being negligible. This effect is highly dependent on the degree of resin infiltration into the demineralized enamel [35, 36]. So, ICON resin seems to have better penetration into demineralized enamel surface than universal adhesive resin does.

ICON resin composition is based on a light-curing low-viscosity resin, composed of mixture of Bis-GMA (bisphenol A glycidyl methacrylate) and triethylene glycol dimethacrylate (TEGDMA), which easily infiltrate the enamel pores and block them [37, 38]. ICON-dry, applied before the ICON resin, contains 99% ethanol, and the addition of ethanol increases the penetration coefficient by decreasing the viscosity and contact angle [39]. Also, hydrochloric acid gel erodes the surface layer more efficiently than 37% phosphoric acid and the self-etching adhesives. ICON's use of longer acid conditioning for 2 min by hydrochloric acid could have resulted in deeper resin penetration, thus recording a lower Δ*E* than universal adhesive resin [40]. As a supporting conclusion to our study, Enan et al. detected that ICON resin could be a potential technique to repair the appearance of demineralized enamel following orthodontic treatment while also protecting it from acidic drinks [41]. The tendency of ICON resin to occlude the pores within the lesion body through infiltration, generating a negligible difference in refractive index with the surrounding sound enamel and increasing the value parameter of the treated lesion, has been concluded by Neuhaus et al. [42]

Conversely, the higher Δ*E* of the universal adhesive resin compared to ICON may be related to its lower penetration coefficient relevant to the weaker self-etching strategy. Thus, the enamel treated with ICON offers a clinically acceptable appearance and brings about patient satisfaction. Combining CPP-ACP with universal adhesive resin seems to generate a harmonizing effect wherein CPP-ACP remineralizes the tooth structure and both act synergistically through infiltrating and occluding the porosity within the demineralized surface, thus improving the esthetic through reducing the refractive index [26–28, 35, 36]. Although no studies have investigated CPP-ACP combined with resin infiltrant for the management of WSLs yet, relevant studies [26–29, 31, 35, 36] assessed independently CPP-ACP and resin infiltration effectiveness in WSLs color improvement and their outcomes could be supportive to this investigation complementary treatment protocol.

Surface microhardness results revealed that CPP-ACP and CPP-ACP coupled with universal adhesive resin were the best management protocols to increase the surface microhardness of WSLs. CPP-ACP proved to have the potential to induce remineralization of enamel, causing white spot lesion regression and high surface microhardness recovery. This is assumed to be relevant to free calcium and phosphate ions that were deposited, thereby stimulating remineralization, maintaining a state of supersaturation with regard to tooth minerals, and discouraging enamel demineralization. Also, its ACP nano-clusters are small enough to access demineralized areas [43, 44]. This is consistent with an additional research, concluding that products containing CPP-ACP have a great tendency to regress enamel white spot lesions due to the incorporation of calcium and phosphate of ACP into the demineralized structure [10, 45]. A different supportive meta-analysis determined that CPP-ACP produces excellent remineralization of WSLs, with high surface microhardness regaining, probably through a remineralizing effect. This indicates that CPP-ACP is verified to be effective for the management of WSLs based on both in-vitro and in-vivo records [46].

The surface microhardness of enamel treated with resin infiltrants is dependent on the degree of enamel demineralization, penetration of the resin, and monomer and solvent compositions of the infiltrants [47, 48]. ICON resin is a Bis-GMA-based infiltrant, and this type of methacrylate resin is known for its high molecular weight, aromatic backbone, and rigid molecular structure. Besides, it contains hydroxyl groups that generate strong hydrogen bonds. All these compositional belongings seem to be responsible for the higher hardness value compared to the preceding demineralized enamel and universal adhesive resin-treated WSLs [49–51]. Contrariwise, the type of resin in the experienced universal adhesive (Gluma Bond) is urethane dimethacrylate (UDMA)-based resin. This resin comprises a flexible aliphatic core with two urethane linkages and is capable of forming hydrogen bonds. Yet, these interactions are not as strong as those in Bis-GMA-based resin. The weaker interactions, plus the flexible nature of UDMA resin, are the causative factors for significantly lower surface microhardness values compared to ICON resin-treated lesions [52–54].

Concerning the penetration coefficient, those infiltrants with a high penetration coefficient would be able to penetrate more deeply into subsurface lesions, filling the spaces between the leftover enamel crystals of the porous lesion and thus enhancing the surface microhardness of the treated lesions [47, 48]. The higher penetration coefficient of ICON resin due to the powerful etching with hydrochloric acid plus the ethanol containing-ICON dry step before ICON application clarifies the difference in surface microhardness values between ICON resin and the universal adhesive resin-treated lesions [39, 55, 56]. Omar El Meligy et al. concluded that ICON resin increased the hardness of demineralized enamel and the stability of carious lesions, relying on its ability to penetrate the porosity of the WSLs, and this finding is consistent with our result [57]. Another investigation confirmed the ability of ICON resin to restore the surface microhardness of the WSLs to a value close to that of sound enamel [58]. In parallel with our findings, another study revealed that both CPP-ACP and ICON resin therapies enriched the tooth surface mineral content, with the enamel mineral gain potential of CPP-ACP being superior [59]. One more in-vitro long-term study disclosed that both ICON resin and CPP-ACFP (casein phospho peptide amorphous calcium fluorophosphate) were effective in treating WSLs, but the CPP-ACFP was more effective as it was extended for a longer period, producing stabilized remineralization of the lesion [60].

While the universal adhesive resin alone was not as efficient as ICON resin in regaining the surface microhardness of the WSLs, pairing it with the CPP-ACP remineralizing agent appeared to complement one another and produce a superior impact on the lesion microhardness compared to other considered approaches. This may be due to the remineralizing competency of CPP-ACP augmented by the resin penetration within the porosity left after remineralizing agent application [35, 36, 43, 44]. An extra contributing factor should be considered, which is the acidic functional monomer; 10-methacryloyloxydecyl dihydrogen phosphate incorporated in the universal adhesive, which generates surface micro-retention and chemical interactions with calcium ions either in the tooth hydroxyapatite or those deposited by CPP-ACP [22].

For CPP-ACP, ICON resin, universal adhesive resin, and CPP-ACP conjugated with universal adhesive-demineralized enamel-treated groups, they were all significantly different from artificially-created WSLs with reference to the surface roughness. CPP-ACP proved to have an excellent ability to smoothen the enamel surface and promote the esthetic. The deposited calcium and phosphorus ions restored the central areas of enamel prisms gradually until the surface became flat and smooth [56]. This consequence is supported by multiple previous analyses, which indicate that CPP-ACP significantly decreases the surface roughness of enamel by the creation of a layer filling the interprismatic cavities and partially covering the enamel prisms [26, 46, 61, 62]. However, our study finding disagreed with Bayram et al. who concluded that CPP-ACP increased the surface roughness of stripped enamel [63].

In harmonization with other studies, the ability of ICON resin to retain a smoother surface of demineralized enamel was confirmed [41, 64]. Other investigations conflicted with this opinion and reported that the surface roughness of resin-infiltrated enamel was less than ideal [65, 66]. The inability of ICON resin to decrease surface roughness was confirmed by an investigation, suggesting that ICON resin increases plaque accumulation in the proximal area, rendering higher surface roughness values compared to pre-application [57]. Also, Gurdogan et al. disagreed with the potentiality of ICON resin to generate a smoother surface when it infiltrates demineralized enamel surface, where they used sound bovine incisors (smooth buccal surface) in contrast to our study, where sound human premolars were investigated [67].

Universal adhesive resin is a flexible cross-linked urethane-based resin with higher mobility than Bis-GMA-based resin. It infiltrates the enamel and leaves a resin layer on the top responsible for the smoothing of the demineralized enamel surface [68]. This may be in contrast to another study that compared ICON to UDMA-based infiltrant and concluded that the UDMA-based resin significantly lowered the surface roughness of demineralized enamel compared to ICON [57]. The CPP-ACP conjugated with the universal adhesive resin-treated group had a statistically comparable result, supposed to be attributed to occluding the pores within the WSL and smoothening of the extremely rough demineralized enamel surface.

Coupling the CPP-ACP remineralizing program with subsequent universal adhesive resin infiltration may be an effective treatment for orthodontic post-bonding WSLs. Investigating the effectiveness of the tested protocol, CPP-ACP coupled with universal adhesive resin, when applied pre-bonding of appliances as a preventive strategy, is compulsory. Regarding the methodology, the recommended protocol seems to be easy to be applied in-vivo. However, the clinical application and success may be highly reliant on the patient's compliance, cooperation, and patience as the patient has to apply the CPP-ACP treatment section daily for 6 weeks with monitoring by the clinician. On accomplishment of the remineralizing program, the universal adhesive resin infiltration is applied in one visit with a prompt restoration of the esthetic.

The current study has some limitations that need to be acknowledged. The limited sample size, the use of artificially-created white spot lesions, and the short-term assessment of the investigated protocol are the most noted restrictions of this study.

Further supporting studies are highly recommended to assess the efficacy and long-term stability of the proposed treatment protocol, CPP-ACP remineralizing agent with subsequent universal adhesive resin, particularly in vivo for the naturally occurring white spot lesions. Additionally, testing this protocol as an effective preventive approach before fixation of the orthodontic appliances should be considered.

## Conclusions

Depending on the findings and within the study's constraints, the following conclusion could be drawn:

- ICON resin and CPP-ACP conjugated with universal adhesive were the best strategies to restore the esthetic of WSLs and generated a statistically comparable consequence, although CPP-ACP conjugated with universal adhesive had a lower value.
- CPP-ACP alone and the CPP-ACP remineralizing program followed by universal adhesive resin infiltration were the best approaches to recover the surface microhardness of WSLs.
- All tested protocols, ICON resin, CPP-ACP remineralizing agent, universal adhesive resin, and CPP-ACP coupled with universal adhesive resin were effective in developing a smoother surface of the WSLs.

## Data Availability

All data presented or analyzed during this study are included in this article and are available from the corresponding author on reasonable request.
